# Animal Models, Learning Lessons to Prevent and Treat Neonatal Chronic Lung Disease

**DOI:** 10.3389/fmed.2015.00049

**Published:** 2015-08-07

**Authors:** Alan H. Jobe

**Affiliations:** ^1^Division of Pulmonary Biology, Cincinnati Children’s Hospital Medical Center, University of Cincinnati, Cincinnati, OH, USA

**Keywords:** prematurity, lung injury, inflammation, oxidant injury

## Abstract

Bronchopulmonary dysplasia (BPD) is a unique injury syndrome caused by prolonged injury and repair imposed on an immature and developing lung. The decreased septation and decreased microvascular development phenotype of BPD can be reproduced in newborn rodents with increased chronic oxygen exposure and in premature primates and sheep with oxygen and/or mechanical ventilation. The inflammation caused by oxidants, inflammatory agonists, and/or stretch injury from mechanical ventilation seems to promote the anatomic abnormalities. Multiple interventions targeted to specific inflammatory cells or pathways or targeted to decreasing ventilation-mediated injury can substantially prevent the anatomic changes associated with BPD in term rodents and in preterm sheep or primate models. Most of the anti-inflammatory therapies with benefit in animal models have not been tested clinically. None of the interventions that have been tested clinically are as effective as anticipated from the animal models. These inconsistencies in responses likely are explained by the antenatal differences in lung exposures of the developing animals relative to very preterm humans. The animals generally have normal lungs while the lungs of preterm infants are exposed variably to intrauterine inflammation, growth abnormalities, antenatal corticosteroids, and poorly understood effects from the causes of preterm delivery. The animal models have been essential for the definition of the mediators that can cause a BPD phenotype. These models will be necessary to develop and test future-targeted interventions to prevent and treat BPD.

## Introduction

**Figure 1:**
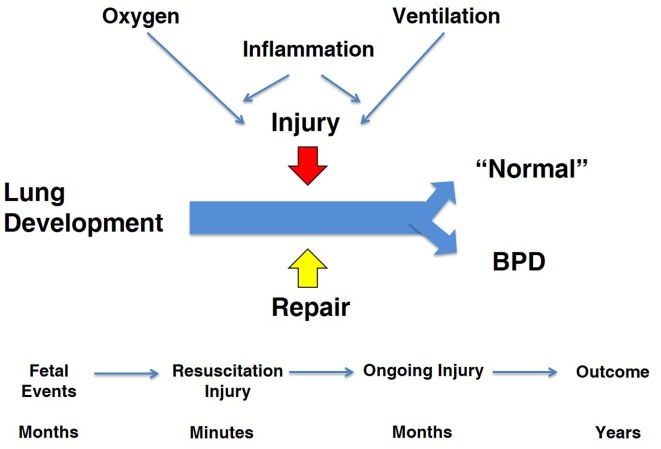
**Overview of “big events” in pathophysiology of BPD**. The three major programs that are active during the course of BPD are lung development, injury, and repair. The major injuries are from oxygen and ventilation-associated inflammation. The time line for the fetus and infant occurs over months to years, which contrasts to studies in term rodent models.

My charge is to evaluate the contributions from animal models that have resulted in better care to prevent and treat chronic lung disease in newborns, which I will refer to as bronchopulmonary dysplasia (BPD). I will focus exclusively on the BPD associated with very preterm birth (1). This review will move between observations in animal models and the clinical syndrome of BPD with a skeptical eye toward how results from models have translated to clinical outcomes. The cynic might say that there has been no progress in the care of infants with BPD based on a recent systematic review of randomized controlled trials (RCT) for the prevention of BPD (2). Of 47 RCTs for drug therapies, only eight showed benefits for five agents (vitamin A, caffeine, dexamethasone, inositol, and clarithromycin) and none of the 47 trials were registered for an Investigational New Drug for BPD. This depressing perspective is in part the result of the lack of trial data to document the continuing improvements in respiratory support and general clinical care that have strikingly decreased mortality for preterm infants since 1967. For example, the two major interventions of surfactant and antenatal corticosteroid treatments decrease the incidence and severity of respiratory disease syndrome (RDS) and decrease mortality after very preterm birth (3, 4). The collateral damage of increased survival is that more high infants are at risk of developing BPD. Antenatal corticosteroids and surfactant change the population of infants at risk for BPD but do not decrease the incidence of BPD. As the disease has evolved to primarily occur in the most immature infants, the concepts about pathophysiology depend more on studies of developing lungs in preterm and term animals. This review will focus primarily on models of BPD in term newborn and preterm animals. My perspective of the pathophysiology of BPD is that the programs required for normal lung development from the saccular to the alveolarized lung are clashing with prolonged exposures to lung injuries from oxygen toxicity, mechanical stretch, and inflammation and simultaneously with repair programs to yield a most complex and varied pathophysiology (Figure 1). Each of the three elements in the equation – development, injury, and repair – is crucial to anticipating how an animal model might contribute to understanding the pathophysiology of BPD and to the testing of treatment strategies.

## An Historical Perspective on Animal Models of BPD

### Oxygen and BPD

Soon after the description of BPD as a progressive lung injury in ventilated premature infants exposed to at least 150 h of 80–100% oxygen (5), Northway’s group reported that newborn mice survived for weeks in 100% oxygen with only a small increase in lung wet weights (6, 7). In contrast, adult mice died within several days and had heavy and inflamed lungs. The histopathology in the newborns demonstrated type II cell proliferation and a hypertrophic bronchiolar epithelium. This increased survival of newborns occurred despite low levels of the antioxidant enzymes that ostensibly should put the newborn lung at increased risk of injury (8). Nevertheless, the most frequently used animal model for evaluating therapies for BPD is the newborn rodent – rats or mice – exposed to various amounts of oxygen for periods of days to weeks as reviewed by Hilgendorff et al. (9).

These rodent models are attractive as they are reproducible, readily available, and relatively inexpensive. Oxidant injury of the newborn rodent lung delays saccular septation to alveoli, increases mesenchymal cellularity, causes a persistent inflammatory response, blocks capillary formation in the distal lung, and causes pulmonary hypertension (10). These changes are similar to the abnormalities described for the lungs of infants who have died of BPD after several months of life (11). Oxygen toxicity models are deceptively simple and are assumed to capture the key elements of the pathophysiology of BPD. However, multiple variables of relevance to BPD can modulate oxygen toxicity responses. As noted above, high concentrations of oxygen kill adult rodents within about 3 days but newborns can survive for weeks in 100% oxygen (6). This transition to oxygen sensitivity occurs after about 30 days of age in the rat (12). Calorie restriction of pregnant rats or newborn rabbits increases the oxygen sensitivity of the lung (13). In adult rats and mice, a variety of exposures prior to an exposure to 100% oxygen can greatly decrease mortality from oxygen – the phenomenon of preconditioning. For example, adult rats exposed to 100% oxygen for 48 h and then returned to air for 24 h prior to a second exposure to 100% oxygen survived for 7 days (14). A pre-exposure of adult rats to endotoxin survive for >3 days in oxygen (15). Fetal sheep exposed to antenatal corticosteroids or to intra-amniotic endotoxin have large increases in antioxidant enzymes in the fetal lungs (16, 17). Many infants at risk of BPD have been exposed to intra-amniotic infection, corticosteroids, and nutritional deficits. Further, hypoxia is a potent preconditioning exposure (18). The assumption generally is that extremely low birth weight (ELBW) infants are very sensitive to oxygen toxicity. However, the animal model data suggest that ELBW infants may have quite variable sensitivities to oxygen at different times during their clinical course because of their antenatal and postnatal exposures. There is no information about oxygen sensitivity in ELBW infants or how that might be assessed (19).

### Ventilation and BPD

In 1960s, mechanical ventilation of large preterm infants with RDS with primitive ventilators and no positive end expiratory pressure (PEEP) resulted in mortality rates of about 70% worldwide. In that context, Northway and colleagues (5) hypothesized that the combination of very high and prolonged oxygen exposure, together with mechanical ventilation, caused the new syndrome of BPD. The initial animal models explored the oxygen exposure component of the injury and no clinical progress was made with ventilation strategies until Gregory and colleagues in 1971 (20) described continuous positive airway pressure (CPAP) to open the atelectatic and surfactant deficient lungs of infants with RDS which improved oxygenation and survival. CPAP was quickly designed into infant ventilators to provide PEEP. The respiratory care of the larger infant with RDS was transformed within several years without much in the way of clinical trials or animal-based research. However, smaller and more immature infants now survived with a new variant of BPD (11).

Animal models then became critical for understanding the link between mechanical ventilation and injury of the preterm lung. Robertson and colleagues developed techniques for short-term ventilation of preterm rabbits, primarily to test physiological responses to surfactant treatments (21). They described severe bronchiolar epithelial disruption within minutes of ventilation of surfactant deficient preterm rabbits (22). The epithelial injury resulted in airspace flooding with intravascular proteins, which could be prevented with surfactant treatment (23). Escobedo et al. (24) and Coalson et al. (25) ventilated premature baboons with oxygen and described chronic BPD-like lung changes as a model to explore the pathogenesis of BPD. This group related the progression of “diffuse alveolar damage” from the combined exposures of oxygen and mechanical ventilation to similar studies in adult baboons with adult respiratory distress syndrome (ARDS) (26). The exudative response to the injury was delayed and blunted in the preterm relative to the adult, a result consistent with less injury from oxygen. However, airway injury was more severe in the preterm lungs. The review of BPD by O’Brodovich and Mellins (27) in 1985 identified oxygen as the primary instigator of BPD, although mechanical ventilation was recognized as a contributor.

**Figure 2:**
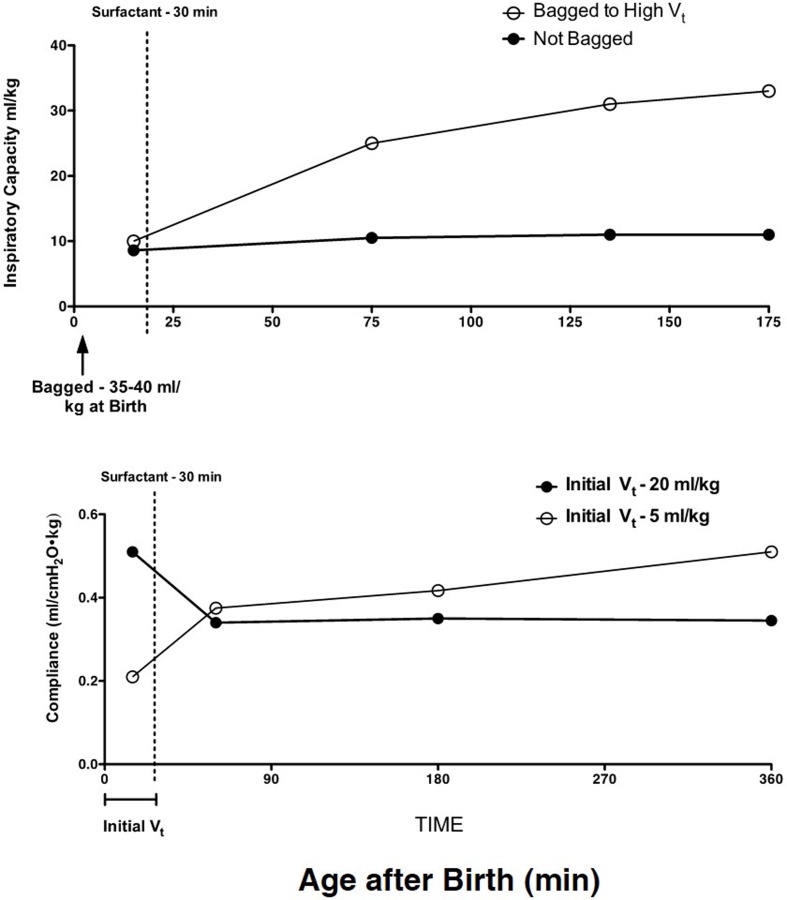
**High tidal volumes inure the preterm lung**. Six large tidal volumes given by hand at delivery injure the preterm lamb lung and prevent a response to surfactant given at 30 min of age. Redrawn from data in Ref. (31). B. An initial tidal volume of 20 ml/kg results in a high compliance in preterm lambs relative to a low tidal volume of 5 ml/kg, but at 30 min of age, the surfactant response is lost with the high tidal volume. The low tidal volume to 30 min of age allows the lungs to respond to surfactant. Redrawn from data in Ref. (32).

Concurrently, with extensive adult animal model research on the mechanical causes of lung injury (28, 29), short-term studies with preterm sheep demonstrated that the preterm lung was exquisitively sensitive to lung injury from volutrauma (30). With the initiation of ventilation of the fluid-filled lung of the preterm lamb with just six very large tidal volumes of 35–40 ml/kg, the preterm lung was injured such that there was no response to surfactant treatment (31) (Figure 2). Tidal volumes of 20 ml/kg for 30 min of ventilation resulted in better compliance and gas exchange than tidal volumes of 5 or 10 ml/kg, but the higher tidal volume injured the lungs (32). This result is similar to the outcomes of tidal volume ventilation of ARDS patients: a higher tidal volume resulted in better gas exchange in the short-term but more mortality from lung injury (33).

The association of both mechanical ventilation and oxygen with BPD in infants and premature animal models is inevitable as both the infants and the animals generally require both to survive. Using short-term fetal mechanical ventilation with air in sheep, O’Reilly et al. (34) reported injury and bronchiolar remodeling over 7 days. Hillman et al. (35) of ventilated exteriorized fetal sheep for 15 min with 100% nitrogen or 100% oxygen and returned the animals to the uterus for 3 h. Expression of response genes and cytokines demonstrated large injury responses but no differences between the nitrogen- and oxygen-exposed lungs. With chronic ventilation of preterm lambs for 3 weeks using oxygen concentrations <30%, Bland et al. (36) reported increased and abnormally distributed elastin and reduced expression of growth factors that regulate alveolarization. Innovative experiments with ventilated newborn mice for 7 h with 40% oxygen or room air disrupted elastin synthesis and increased apoptosis (37). A subsequent study with 24 h air ventilations of newborn mice demonstrated increased apoptosis and an inhibition of alveolarization and microvascular development, the key findings in BPD (38). These effects were remarkable in that the ventilation induced the BPD phenotype quickly and without a concurrent oxygen exposure or inflammation. In contrast to newborn mice, room air may be “hyperoxic” for the very preterm human lung and is thus an unavoidable exposure. Nevertheless, these experiments with mice in the saccular phase of lung development are proof of principle that mechanical ventilation alone can cause a BPD phenotype.

Lung injury from mechanical ventilation has progressed from considerations of pressure (barotrauma) to focused analyses of tidal volumes (volutrauma), atelectotrauma (ventilation from low lung volumes), and biotrauma (proinflammatory mediators), primarily using adult animal models (39, 40). Strategies to decrease ventilator-induced lung injury (VILI) are major components of efforts to improve outcomes for patients with ARDS (39). VILI was recently compared in adults, children, and with an analysis of animal models (41). Newborn rodents have larger lung volumes [functional residual capacity (FRC) and total lung capacity (TLC)] than adult rodents, which seems to translate to less injury at a given tidal volume in the newborn than for adult animals (42, 43). However, the term newborn rodent is not a good model for the preterm infant at risk of BPD. The preterm infant has a low FRC and a low TLC compared with the term infant or the adult human (30). Further, the surfactant-deficient preterm lung requires mean airway pressures > 20 cmH_2_O to maximally recruit lung volume (44). The preterm lung is easier to overstretch because the chest wall is compliant and the lung collagen matrix is insufficient to limit stretch of both the airways and the parenchyma (45). The lung volume available between FRC and TLC for safe mechanical ventilation may be as low as 15 ml/kg. Thus, with an unstable FRC and a poorly defined TLC, VILI can easily occur with mechanical ventilation. The animal models most relevant for understanding how ventilation injury contributes to BPD are preterm sheep or primates who can be ventilated for prolonged periods so that injury progression can be evaluated.

### Inflammation and BPD

**Figure 3:**
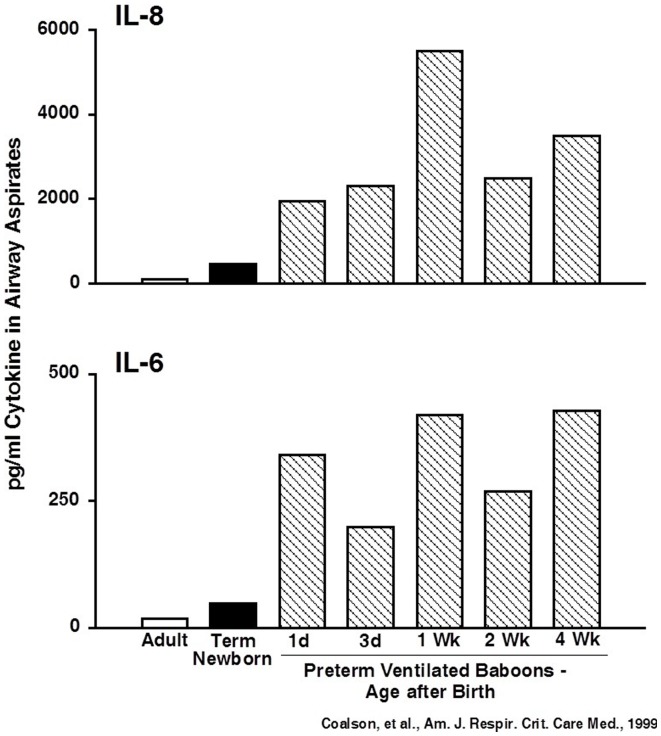
**Cytokines are persistently elevated in airway samples from ventilated preterm baboons**. IL-6 and IL-8 were increased from day 1 to week 4 in preterm baboons progressing to BPD. Data redrawn from Coalson et al. (48).

A common theme since the early days of BPD research is the association between inflammation and BPD. Airway aspirates from infants with early lung injury that progresses to BPD contain increased numbers of inflammatory cells and proinflammatory cytokines (46, 47). As in adult animal models with high oxygen exposures alone, newborn rodent lungs respond to injury with recruitment and activation of inflammatory cells and increased proinflammatory cytokines in alveolar washes (10). Similarly, stretch-mediated injury causes a brisk proinflammatory response in preterm sheep (35). Very preterm baboon lungs developing BPD from oxygen and mechanical ventilation have greatly elevated proinflammatory cytokines for the 4-week period of study (48) (Figure 3). The preterm fetal sheep lung has a large inflammatory response to just 15 min of stretch from ventilation with nitrogen using high (15 ml/kg) or to relatively normal tidal volumes of 6 ml/kg (49, 50). The inflammatory response is less with the lower tidal volume, with antenatal corticosteroid treatment, with surfactant treatment prior to ventilation, or with ventilation with PEEP (51–53). However, even combinations of approaches will not eliminate an inflammatory response with ventilation of the preterm fetal lung. Further, the 15 min lung stretch injury is greatly amplified if the preterm fetus is subsequently delivered and ventilated for brief periods. These experiments in baboons and sheep demonstrate that the preterm lung can generate rapid and very prolonged inflammatory responses to care elements that most ELBW infants need for survival.

However, the clinical environment is much more complex as more than 50% of ELBW infants will have been exposed to infection/inflammation from chorioamnionitis prior to delivery (54). An increased risk of BPD may be associated with fetal exposure to histologic chorioamnionitis, although the increased risk has been inconsistent in the multiple evaluations of the association (55). Watterberg et al. (56) made the intriguing observation that preterm infants exposed to histologic chorioamnionitis had a decreased risk of RDS but an increased risk of BPD. The infants exposed to chorioamnionitis had increased amounts of inflammatory mediators and cells in airway aspirates collected shortly after birth. Therefore, the lung inflammation proceeded the delivery and subsequent exposures to oxygen and ventilation. The difficulty with a consistent interpretation of the clinical data is that chorioamnionitis is caused by organisms ranging from commensals to pathogens, the fetal exposure is for various periods prior to delivery, and the intensities of the exposures are variable (57).

Animal models have been effectively used to characterize the ranges of fetal lung responses to inflammatory exposures. Bry et al. (58) first reported that intra-amniotic injections of interleukin 1 (IL-1)-induced lung maturation in preterm fetal rabbits as measured by increased mRNA for surfactant proteins, increased surfactant lipids, and improved pressure–volume curves. Intra-amniotic injections in fetal sheep of IL-1 or *E. coli* endotoxin lipopolysaccharides (LPS), but not IL-6, IL-8, or TNF-α, induce chorioamnionitis and early lung maturation as measured by large increases in pressure–volume curves and lung mechanics and gas exchange within 5 to 7d (59–61). The improvement in lung performance is proceeded by increases in mRNA for the surfactant proteins and then by increases in surfactant lipids (62). The clinical phenotype consistent with these early maturational effects would be a decrease in the severity of RDS, which should decrease the risk of BPD.

The maturation response to fetal exposure to chorioamnionitis induced by IL-1 or LPS is proceeded by modest inflammation characterized by recruitment of activated granulocytes to the fetal lung parenchyma and airspaces, high levels of proinflammatory cytokine expression, increased apoptosis, and subsequent cell proliferation (63). This injury response includes a transient inhibition of alveolar septation and causes pulmonary vascular injury with increased smooth muscle in vessel walls and decreased mediators of vascular development (64, 65). The fetal lung has more functional maturation and more antioxidant enzymes but with septation and microvascular injuries consistent with a mild BPD phenotype. Despite prolonged exposure to intrauteral inflammation, infants have not been described as having BPD at birth. This observation is consistent with the animal models, as repetitive or continuous exposures for 28 days to intra-amniotic LPS do not cause progression of the septation and microvascular injuries (66). In fact, the preterm lung heals despite continued exposures to proinflammatory agonists. The proinflammation is downregulated soon after exposure to agonists, and the fetal lung “tolerizes” in that subsequent inflammatory mediator exposures do not incite inflammation in the fetal lung (67). LPS is the agonist most used in animal models to evaluate effects on the fetal lung. LPS inhibits structural development of the fetal mouse lung via TLR4 receptors and NF-kB activation with suppression of FGF-10 (68, 69). Mediators such as TGF-β1 and the SMADS are induced while others such as connective tissue growth factor (CTGF) and caveolin-1 are suppressed, demonstrating multiple effects on LPS on the fetal lung that may contribute to the changes of BPD (70, 71).

Multiple different organisms and polymicrobial infections have been associated with histologic chorioamnionitis, but different clinical presentations have not been associated with the types of infection (57). In the fetal sheep or monkey, quite distinct responses of fetal exposures to organisms and mediators can be demonstrated. Intra-amniotic LPS or IL-1 causes limited but very complex inflammatory responses that yield the phenotypes of lung maturation, a mild BPD, and immune tolerance (67, 72). Ureaplasma species are the most frequent organisms associated with preterm labor and they may cause chronic and indolent colonization (infection?) and have been associated with BPD (73). Ureaplasma readily colonize the amniotic fluid of fetal sheep and cause a generally mild but variable chorioamnionitis that can persist for months (74). The fetal lung has a very modest inflammatory response with low-grade cytokine expression and a small increase in granulocytes (75). However, the induced lung maturation and microvascular changes are reminiscent of LPS effects (76, 77). In contrast, intra-amniotic *Candida albicans* causes a modest chorioamnionitis but a severe consolidating and necrotizing pneumonia with a progressive lethal fetal inflammation within about 3 days (78). These varied responses in animal models provide the insight that chorioamnionitis may initiate BPD by decreased septation and microvascular injury in fetal life but may also protect from BPD by inducing lung maturation and increasing antioxidant enzymes. As recently reviewed by Wright and Kirpalani (79), attempts to target inflammation have not translated to therapies for BPD.

## Animal Models to Evaluate Therapies for BPD

### Overview

The major elements of BPD that have been targeted for therapies are the arrest of alveolarization, the blunted microvascular development, and the inflammation that is generally considered a common path to the septation and vascular abnormalities. The concept that inflammation was a common mediator for the progression of BPD resulted from the observations that inflammation accompanied oxygen toxicity and ventilation-mediated injury in newborn and adult animals and that overexpression of most any proinflammatory cytokine in newborn mice lungs resulted in inflammation and septation abnormalities (11). Early examples of a BPD phenotype were overexpression of TGF-α, IL-11, and IL-6 (80–82). More recently, overexpression of IL-1β or bioactive TGF-β1 in airway epithelial cells in newborn mice were proposed as models of BPD (83, 84). The experimental strategy to assess a therapeutic benefit has generally been to cause a BPD-like injury with oxygen exposure of newborn rodent lungs and to assess agonists or antagonist effects on inflammation and changes in septation and/or microvascular development. Primate and sheep models of combined ventilation and oxygen exposure have been used to better replicate clinical reality and to test interventions such as high-frequency ventilation (HFV) and inhaled nitric oxide (iNO). I will assume that newborn rat and mouse models of oxygen exposure are equivalent and I will not address stem cell therapies. In general, the reports focus on individual mechanisms and pathways, but many of the interventions may have pleotropic (and presently unknown) effects.

### BPD therapy by blocking inflammatory cells

If BPD is caused by chronic inflammation in the developing lung, then blocking the major inflammation factories – the granulocytes and macrophages should be effective. Auten et al. (85) demonstrated that antibodies to the cytokine – induced neutrophil chemoattractant-1 (CINC-1) effectively blocked neutrophil recruitment to the lungs and alveolar washes of 95% oxygen-exposed newborn rats and alveolar architecture was preserved. This observation was extended to demonstrate that blocking the signaling of CINC-1 through its receptor CXC chemokine receptor-2 with a small molecular weight antagonist completely blocked neutrophil influx into the lungs of 60% oxygen-exposed newborn rats (86). Surprisingly, the 60% oxygen exposure, together with blocked neutrophil influx, enhanced lung growth and septation. Treatment of newborn rats with 60% oxygen and IL-1 receptor antagonist blocked neutrophil recruitment to the lungs and improved alveolar septation with less of an effect on the pulmonary vascular injury (87). Neutrophil recruitment to the lungs of fetal sheep exposed to LPS can be effectively blocked by either an anti-CD-18 antibody or by intra-amniotic IL-1 receptor antagonist (88, 89). Either strategy to decrease inflammatory cell recruitment to the fetal lungs prevents both the injury and the lung maturation responses. Strategies to interfere with neutrophil recruitment to prevent BPD have not been tried in infants at risk. Such strategies have not been effective for ARDS and infants have depressed immune responses that would be further compromised by inhibition of granulocyte function.

The normal developing lung contains few mature macrophages until after birth when an increase in GMCSF in the lungs activates the transcription factor PU-1 in immature monocytes to differentiate to mature monocytes (90). However, macrophages become prominent in airway samples of infants with BPD. Monocytes in the lungs of fetal sheep exposed to a 15 min volutrauma injury mature within 24 h to macrophages as do fetal lung monocytes exposed to intra-amniotic LPS (90, 91). Jankov et al. (92) used gadolinium chloride to block macrophage influx into the lungs of newborn rats exposed to 60% oxygen. The treatment decreased macrophages and prevented smooth muscle hypertrophy in pulmonary vessels and prevented pulmonary hypertension but not the septation defect – a partial response at best. As with inhibitors of granulocyte recruitment, macrophage depletion may put the preterm at risk of infection.

Targeting overall lung cell metabolism has not been exploited for a possible therapy for BPD. In newborn mice exposed to 75% oxygen, decreased septation correlated with decreased mitochondrial oxidative phosphorylation (93). Thus, a general cellular bioenergetics failure maybe a fundamental mechanism contributing to BPD. Inflammasome-mediated cytokine injury has not been explored in the developing lung. Mechanical ventilation of adult mice activated inflammasomes with IL-18 release, an effect that was blocked with an anti-IL-18 antibody with reduced lung injury (94). These reports may be relevant to future BPD research.

### Antioxidants to prevent BPD

Increased oxygen exposure is considered a major cause of BPD, and oxygen is the agonist for the newborn rodent models of BPD. Lung antioxidant enzymes are decreased with preterm birth (8), so that antioxidant treatments are logical. Clinically, *N*-acetylcysteine and recombinant superoxide dismutase did not decrease BPD (95, 96). Vitamin A, which may have antioxidant effects, did modestly decrease BPD (97). Consistent with the clinical result, Vitamin A improved septation and alveolar capillary growth in chronically ventilated preterm lambs (98). Vitamin A also modulated elastin metabolism and the expression of vascular growth factors as mechanisms distinct from antioxidant effects. In preterm ventilated baboons given 100% oxygen for 10 days, a small molecular weight catalytic antioxidant improved lung anatomy relative to untreated animals (99). Peroxynitrites are products of oxidant exposure, and a catalyst that decomposes peroxynitrite decreased both the alveolarization and the vascular injuries resulting from exposure of newborn rats to 60% oxygen (100, 101). The animal model research with antioxidants for BPD is not extensive and might be a productive avenue toward a therapy.

### Nitric oxided to prevent BPD

The animal model literature on the benefits of iNO for the preterm lung is large and positive. Lambs exposed to mechanical ventilation have decreased neutrophil accumulation and edema if exposed to iNO (102). iNO also attenuates pulmonary hypertension and improves lung growth in newborn rats exposed to hyperoxia (103), as does the related compound ethyl nitrate (104). iNO also corrects the lung structural abnormalities and pulmonary hypertension from a bleomycin-induced lung injury in neonatal rats that resembles BPD (105). iNO can improve the lung structural abnormalities caused by blockade of the vascular endothelial growth factor (VEGF) receptor, suggesting iNO effects on growth factor signaling (106). These beneficial effects translated to chronically ventilated large animal models in preterm lambs and baboons. Bland et al. (107) found that iNO protected ventilated lambs from alveolar simplification and increased airway resistance. iNO treatment of preterm ventilated baboons over 14 days improved lung function, improved septation, preservation of lung growth, and normalization of extracellular matrix deposition (108). As a treatment strategy to mimic NO signaling, Ladha et al. (109) treated newborn 95% oxygen-exposed rats with sildenafil to increase cGMP and improved alveolar growth with less pulmonary hypertension. Another approach used by McCurnin et al. (110) was to give estradiol to increase NO synthases, a treatment that improved lung function of preterm ventilated baboons over 14 days, but without clear effects on lung structure or elastin deposition. Despite this extensive literature in both newborn rodents and chronically ventilated sheep and baboons that identify multiple beneficial effects of iNO, multiple clinical trials show no consistent benefit of iNO for the prevention of BPD in preterm infants (111), a most disappointing result.

### Growth factors in the pathogenesis of BPD

Lung growth from the saccular to alveolar stages of development is regulated by multiple growth factors that may be dysregulated during the course of BPD. In newborn rats, inhibition of VEGF decreases lung septation and microvascular development in parallel (112), a phenotype similar to the oxygen-exposed newborn rodent. Therefore, Kunig et al. (113) exposed newborn rats to 75% oxygen for 14 days and then treated the animals with intramuscular injections of rhVEGF and demonstrated improved recovery of vascular growth and alveolarization relative to controls. A similar result was reported for prevention of the initial oxygen injury in newborn rats (114). The vascular and alveolar development that was inhibited by 95% oxygen did not occur with postnatal adenovirus-mediated VEGF therapy. Optimal treatment included both VEGF and angiopoietin-1 gene transfers to decrease the vascular leakage caused by the increased VEGF expression. Such a complex treatment would be impractical in humans, but the prevention of injury and repair responses of the oxygen-injured rodent lung are proof of principal that VEGF dysregulation likely contributes to clinical BPD.

Other growth factors also have therapeutic benefits in animal models of BPD. Keratinocyte growth factor (KGF or FGF7) can protect newborn rats from death from pulmonary hypertension but not for the decreased septation in >95% oxygen over the first 2 weeks of life (115). Franco-Montoya et al. (116) noted that intraperitoneal KGF prevented neutrophil recruitment to the airspaces of >95% oxygen-exposed newborn rats with less decrease in DNA or cell proliferation, but the structural changes in the newborn lungs were not changed by KGF treatment. Thus, any treatment benefits of KGF in the rodent model were partial at best.

In contrast, hepatocyte growth factor (HGF) had more remarkable effects on newborn rodent lungs exposed to 90% oxygen. Intraperitoneal HGF partially corrected increased airway resistance and hyper-responsiveness, improved the alveolar simplification, and increased the microvasculature (117). Platelet-derived growth factor can have long-term effects on septation if its signaling is disrupted with a receptor antagonist even briefly (118). Therapy with PDGF has not been reported in BPD models. Subramaniam and colleagues (119) report that bombesin-like peptides predict the severity of BPD in ventilated preterm baboons, and an antibody to these peptides improved the septation. Clearly, multiple growth factors act in time and space to regulate lung development. Although animal models support growth factor–based treatments for BPD, caution is warranted as such therapies may have adverse effects on other developing systems.

### Other treatments for BPD in animal models

Glucocorticoid treatments have been used for the prevention of BPD (early treatments) and to treat the progression of BPD (120). Although there are concerns about the pleotropic developmental effects, dexamethasone can decrease the incidence of BPD and mortality. The presumed mechanism of action is as a potent anti-inflammatory. Cyclooxygenase-2 (Cox-2) is upregulated in the 60% oxygen exposure rodent model of BPD and in lungs with BPD (121). Therefore, Masood and colleagues (122) used a selective Cox-2 inhibitor to suppress prostaglandin production during oxygen exposure of newborn rats with remarkable results. The Cox-2 inhibitor prevented recruitment of neutrophils and macrophages and blocked the inhibition of alveolar septation and the increase in tissue fraction caused by oxygen. Choo-Wing et al. (121) used transgenic technology to overexpress interferon-γ and small interfering RNA to inhibit the endoplasmic reticulum stress pathway in hyperoxia-exposed newborn mice. A Cox-2 inhibitor or the siRNA prevented the arrest of alveolarization caused by INFα and hyperoxia. These results suggest inhibition of Cox should be an effective therapy for BPD, but indomethacin or ibuprofen treatments of infants to decrease intraventricular hemorrhage or to close the patent ductus arteriosus are not associated with a decrease in BPD (123). However, specific Cox-2 inhibitors have not been directly tested to prevent BPD.

TGF-β1 is an important mediator of both stimulation and inhibition of lung cell development and airway branching (124). Airspace fluids from infants with BPD contain increased amounts of TGF-β1 and antenatal intra-amniotic LPS greatly induce TGF-β1 in the lungs of fetal lambs (70). Nakanishi et al. (125) demonstrated the alveolar and vascular abnormalities caused by 95% oxygen in newborn mice can be partially prevented with a TGF-β neutralizing antibody.

Focal elastin bundles identify the sites of septation, and inhibition of elastin formation or abnormal distributions of increased amounts of elastin in saccular/alveolar walls inhibit septation. Ventilated and oxygen-exposed preterm lambs have inhibited alveolarization. Lung growth factors (VEGF, PDGF-A) and their receptors were decreased at 3 weeks (36). Elastin-related gene expression increased and discrete septation initiation sites were lost with prolonged ventilation of the preterm lamb. Remarkably, these effects on septation sites to uncouple the synthesis and assembly of elastin can occur within 24 h of mechanical ventilation in newborn mice (37). Using the ventilated newborn mouse model, Hilgendorff et al. (126) then showed that intratracheal treatment with the elastase inhibitor elafin prevented elastin degradation, TGF-β activation, apoptosis, and decreased the septation abnormality. This result was reproduced using newborn mice that overexpressed elafin (126). These experiments demonstrate a therapeutic strategy that simply decreases the elastin degradation that accompanies mechanical ventilation.

Other pathways activated by oxygen or bleomycin exposure of newborn rodents demonstrate both the complexity of the oxygen injury, but also how targeting single pathways can prevent much of the injury. Rho-kinase is a ubiquitously expressed protein that regulates numerous cell functions and proliferation. It is upregulated by bleomycin in the newborn rat, and inhibition of Rho-kinase decreases the BPD-like changes caused by bleomycin (127). B-catenin is another multifunctional signaling protein that is critical for normal lung development and remodeling and aberrant β-catenin signaling has been associated with BPD. In the 90% oxygen-exposed newborn rat model, an inhibitor of β-catenin mitigated the septation defect and pulmonary hypertension (128). A recent observation is that an extract from a Chinese traditional herb *Astragalus membranaceus* has anti-inflammatory effects and protects newborn rats from oxygen injury (129). Finally, infants with BPD often have hypercarbia. Although hypercarbia can have deleterious effects on the lung (130), in the newborn rat hypercapnea blunted the influx of macrophages, and the resulting pulmonary hypertension caused by bleomycin (131). With use of TNF-α inhibitor, the benefits of hypercapnea were explained by fewer macrophages secreting TNF-α.

## Ventilation Strategies to Decrease Lung Injury

### High-frequency oscillatory ventilation

Mechanical ventilation of the premature infant is a major contributor to lung injury that progresses to BPD. A strategy to minimize injury that was initially tested in preterm animals was high-frequency oscillatory ventilation (HFOV). Preterm baboons supported by HFOV required less supplemental oxygen and had fewer air leaks than animals supported with conventional mechanical ventilation (CMV) (132). All HFV animals survived for 11 days while 5 of 11 animals on CMV died within 48 h. In 6 h experiments, Jackson and colleagues (133) reported that premature *Macaca nemestrina* ventilated with HFV had better expanded lungs with less alveolar debris and better gas exchange. Other measures of lung injury were not different. A subsequent report with this primate model demonstrated that HFV when combined with surfactant treatment caused less injury than HFV or surfactant treatments alone (134). Yoder et al. (135) used very preterm baboons at 125 days gestational age (GA) who were exposed to antenatal steroids and treated with surfactant at birth to compare HFV with CV using tidal volumes of 4–6 ml/kg for 1–2 months. Prolonged HFV resulted in better lung mechanics, a lower oxygen requirement, and lower cytokine levels in airway samples at selected timesV when combined to 1 month of age. At autopsy, the HFV animals had better inflated lungs, but both groups had pathologic changes of BPD. Overall the animal studies support substantial benefits of HFV relative to CMV. Unfortunately, meta-analyses of 3,260 randomized infants found no net benefit of HFV for decreasing BPD (136). An individual patient analysis of nine trials to evaluate HFV relative to CMC also showed no BPD benefit for HFV (137). However, a recent report demonstrated better pulmonary function tests at 11–14 years of age for infants randomized to HFC relative to CMV (138). Overall, the animal testing was not predictive of clinical benefit.

### Tidal volumes and continuous positive airway pressure

The preterm lung can be easily injured with high tidal volumes as demonstrated by the effects of high tidal volumes following birth (31, 32). Animal models were used to evaluate chronic effects of tidal volumes and non-invasive ventilation strategies. Preterm surfactant-treated lambs were randomized to 20 breaths/min with 15 ml/kg tidal volumes or 60 breaths/min with 6 ml/kg tidal volumes for 3–4 weeks by Albertine et al. (139). Both ventilation groups had abnormal alveolarization relative to age matched non-ventilated controls. The high tidal volume animals had more impaired alveolarization and more elastin than the low tidal volume animals. Studies of similarly ventilated lambs showed decreased pulmonary microvasculature, interstitial edema and increased pulmonary arteriolar smooth muscle and elastin in both groups of animals (140).

Animal models also were used to test the hypothesis that non-invasive respiratory support would injure the preterm lung less than CMV. 125 days GA baboons were treated with surfactant and stabilized with CMV for 24 h before extubation to nasal CPAP for 4 weeks (141). As compared to fetal controls, the CPAP animals had enlarged thin walled air spaces, but with minimal injury compared to animals supported with CMV. A second study with preterm baboons evaluated an early 24 h extubation to CPAP or a delayed 5 days extubation to CPAP (142). The delayed CPAP group had worse oxygenation and more inflammation and higher proinflammatory cytokine levels in the airway samples than did the early CPAP group, but morphometry for alveolarization was similar between groups. Preterm lambs ventilated using either non-invasive high-frequency nasal ventilation or CMV with tidal volumes of 5–7 ml/kg were evaluated at 3 days for the effects of the ventilation style on mesenchymal cell apoptosis and proliferation (143). The non-invasive ventilation achieved more thinning of the mesenchyme with more apoptosis and less proliferation that supported alveolarization better than the CMV group. As with the HFO studies, these non-invasive ventilation approaches decreased a variety of indicators of lung injury better than CMV. However, comparisons of CPAP strategies with intubation, surfactant treatment, and CMV had only marginal benefits to prevent BPD in four trials in very low birth weight infants (144).

### Preconditioning for BPD-like responses of newborn lungs

The animal models and treatments described so far have used a single lung injury – oxygen, bleomycin, or ventilation – to induce BPD-like changes in developing lungs. Clinical medicine is much more complex because of the multiple associations with preterm delivery. There is a large literature about how an antenatal exposure can modulate a postnatal exposure (18). A representative example is the report by Tang et al. (145) that demonstrates that fetal exposure of mice to intra-amniotic LPS causes a BPD phenotype in the newborn mice. Postnatal exposure to >95% oxygen increases the BPD-like abnormalities. However, postnatal exposure to 65% oxygen has the opposite effect of accelerated lung growth and attenuated pulmonary hypertension. A second example is the competing effects of intracervical *E. coli* and betamethasone on postnatal hyperoxic exposures in rats (146). The hyperoxic responses were increased by the antenatal inflammation but attenuated by the combination of inflammation and betamethasone. A post-delivery exposure such as hyperoxia also can aggravate lung injuries later in life. Newborn mice exposed to 100% oxygen for 4 days and allowed to recover in room air were much more sensitive to bleomycin-induced fibrosis or influenza virus as adults (147, 148). The important message is that one exposure that may cause a BPD phenotype may be substantially modified by a previous or subsequent exposure. These complex exposures can only be evaluated in animal models.

## Summary

The animal models of BPD that use premature sheep, primates, or newborn rodents have injury responses that are similar to what is known about the pathology of BPD. A cautionary note is that the pathology is only available for infants who have died of end stage BPD, with few exceptions. The range of abnormalities may be far greater for the majority of infants who survive BPD to remodel and grow their lungs. The interventions in animal models to decrease oxidant injury, inflammation, and volutrauma each show rather striking benefits for the major pathologies of decreased septation and microvascular development. Given the list of positive strategies enumerated in this review, effective treatments should be either in use or in late stages of development. But they are not.

My explanation is that the animals are studied as normal fetuses or newborns, and virtually all preterm infants are abnormal because of the abnormalities associated with very preterm delivery, primarily placental dysfunction, fetal exposure to infection, and fetal growth restriction (149). Further, >80% of the infants have been exposed to antenatal corticosteroids (1). Preconditioning events likely change each infant’s responses to oxygen, ventilation, and inflammation. The clinical responses also are likely to be modulated by the genetic variability in humans that is greatly minimized in the animal models. For example, clinical interventions that have been extensively trialed such as antenatal corticosteroids and surfactant treatments required several hundred humans to be randomized to show clinically relevant effects such as decreased mortality (3, 4). Experiments with sheep or primates with group numbers of four to eight can show highly significant differences in physiological outcomes such as lung compliances or oxygenation. Effects of antenatal steroids or surfactant on BPD are minimal in human trials because of decreased mortality with more high-risk infants surviving.

However, the animal models of BPD have shaped our understanding of the disease. Care strategies have improved which has allowed the survival of smaller and more fragile infants. Less aggressive approaches to ventilation, better nutrition, and better control of postnatal infection are hard to document, but in my view are of substantial benefit to preterm infants. Finally, a major deficiency is the clinical definition of BPD that is based on a therapy – supplemental oxygen. The diagnosis does not directly inform the clinician about how the lungs are working or what the abnormalities may be. It may be most difficult to evaluate a therapy based on animal models if there is not more precision in knowing what components of BPD the therapy should improve. Therapies targeting specific pathways that are effective in animal models are most difficult to translate into a safe and effective therapy in preterm infants because of constraints on clinical research in this population. However, animal models will have a critical role in refining our understanding of the pathophysiology of lung injury and repair in the future. Research strategies should include more complex models with multiple and sequential fetal and/or newborn lung injuries. A focus on repair mechanisms may be more informative than studies of acute injury mechanisms. The identification of biomarkers that can be used to identify primary injury or repair pathways may help in the testing of treatments in infants, as surrogate indicators are preferable to long-term outcomes for studies in infants.

## Conflict of Interest Statement

I have consulted about therapies for RDS and BPD with Chiesi, Parma Italy and Abbvie, Chicago, USA. Chiesi has provided surfactant and Fisher & Paykel, Auckland, NZ, has provided supplies for studies of lung injury in preterm sheep performed in Australia. This research was supported by grants from the US National Institute of Health. There was no support for this work nor do I have patents or financial holdings that are related to this work.
